# 11‐year‐old girl presenting with blurred vision and progressive right arm weakness

**DOI:** 10.1111/bpa.70097

**Published:** 2026-03-25

**Authors:** Trung Toan Tran, Thi My Hanh Luong, Bérengère Dadone‐Montaudié, Hong Nhung Ta, Thai Hoc Nguyen, Fanny Burel‐Vandenbos, Gia Du Pham

**Affiliations:** ^1^ Department of Pathology and Molecular Biology Pathology and Molecular Biology Center, Vietnam National Cancer Hospital Hanoi Vietnam; ^2^ Laboratoire Central d'Anatomie Pathologique, Department of Pathology and Molecular Oncology University Hospital of Nice Nice France; ^3^ Tumor Immunopathology Unit Research Institute for Community Medicine Development Hanoi Vietnam; ^4^ Laboratoire d'Oncologie Moléculaire, Department of Pathology and Molecular Oncology University hospital of Nice Nice France; ^5^ Department of Radiology Vietnam National Cancer Hospital Hanoi Vietnam; ^6^ Department of Neurosurgery Vietnam National Cancer Hospital Hanoi Vietnam

BOX 1 **Virtual glass slide.**

Access at https://isn‐slidearchive.org/?col=ISN&fol=Archive&file=BPA‐6927662.svs

## 1 CLINICAL HISTORY

An 11‐year‐old girl, with no significant medical history, presented to the Vietnam National Cancer Hospital with blurred vision in both eyes and progressive weakness in the right arm over a two‐week period. Brain magnetic resonance imaging (MRI) revealed a well‐defined, solid and cystic lesion in the left parietal hemisphere, measuring 53 × 57 mm, causing a significant mass effect (Figure 1). Whole‐body imaging showed no evidence of lesions elsewhere. Intraoperatively, the tumor was entirely intraparenchymal, with its base infiltrating the motor white matter. The solid portion was resected, and the cystic part preserved due to adherence to critical tracts.

**FIGURE 1:**
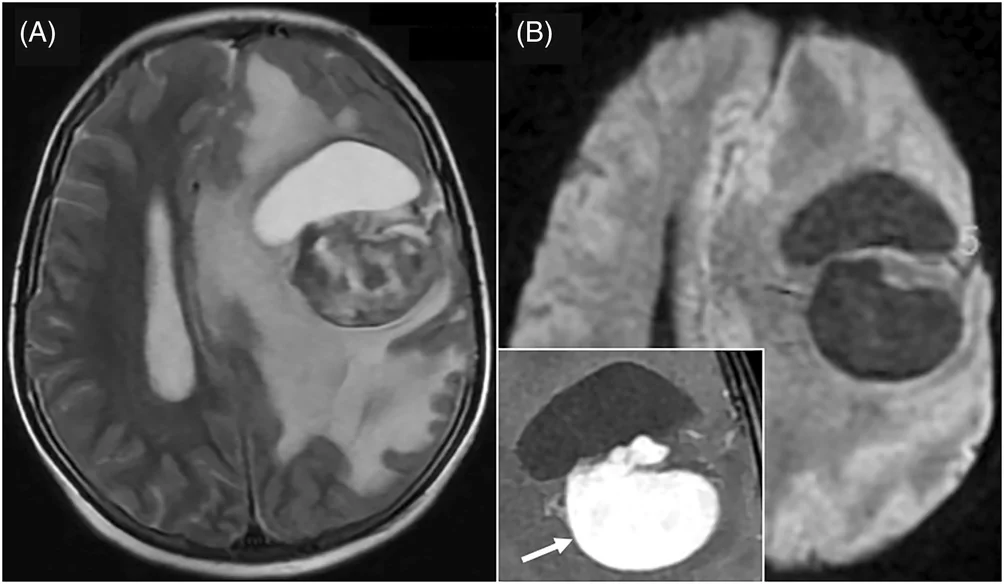
MRI findings. A lobulated, cystic and solid lesion with extensive perilesional edema and mass effect was identified in the left frontal lobe parenchyma. The solid component appeared heterogeneously hyperintense on T2‐weighted images (A), showed no diffusion restriction on diffusion‐weighted imaging (B), and demonstrated intense, homogeneous enhancement on post‐contrast images (inset).

## 2 FINDINGS

Histopathology showed a proliferation of fibroblast‐ and myofibroblast‐like cells with heterogeneous cellularity (Figure 2A,B), vesicular nuclei, small nucleoli, and pale eosinophilic cytoplasm (Box 1). Scattered ganglion‐like and pleomorphic cells were observed (Figure 2C,D), while mitoses were rare. The stroma contained macrophages, lymphocytes, and plasma cells, with focal myxoid changes. Diagnoses of glioma, meningioma, and solitary fibrous tumor were ruled out, based on the negativity of GFAP, OLIG2, EMA, PR, and STAT6. ATRX and INI1 were maintained. CD68 was positive, but Langerin, CD1a, and S100 were negative, excluding histiocytosis. The only other positive immunostainings were CD99, ALK1, and actin (Figure 2E,F), which rather pointed toward the group of mesenchymal tumors. Proliferation index KI67 was 5%. RNA sequencing identified a TMP4::ALK fusion. No additional mutations were detected by DNA sequencing (Ion AmpliSeq Cancer Hotspot Panel, Life Technologies).

**FIGURE 2:**
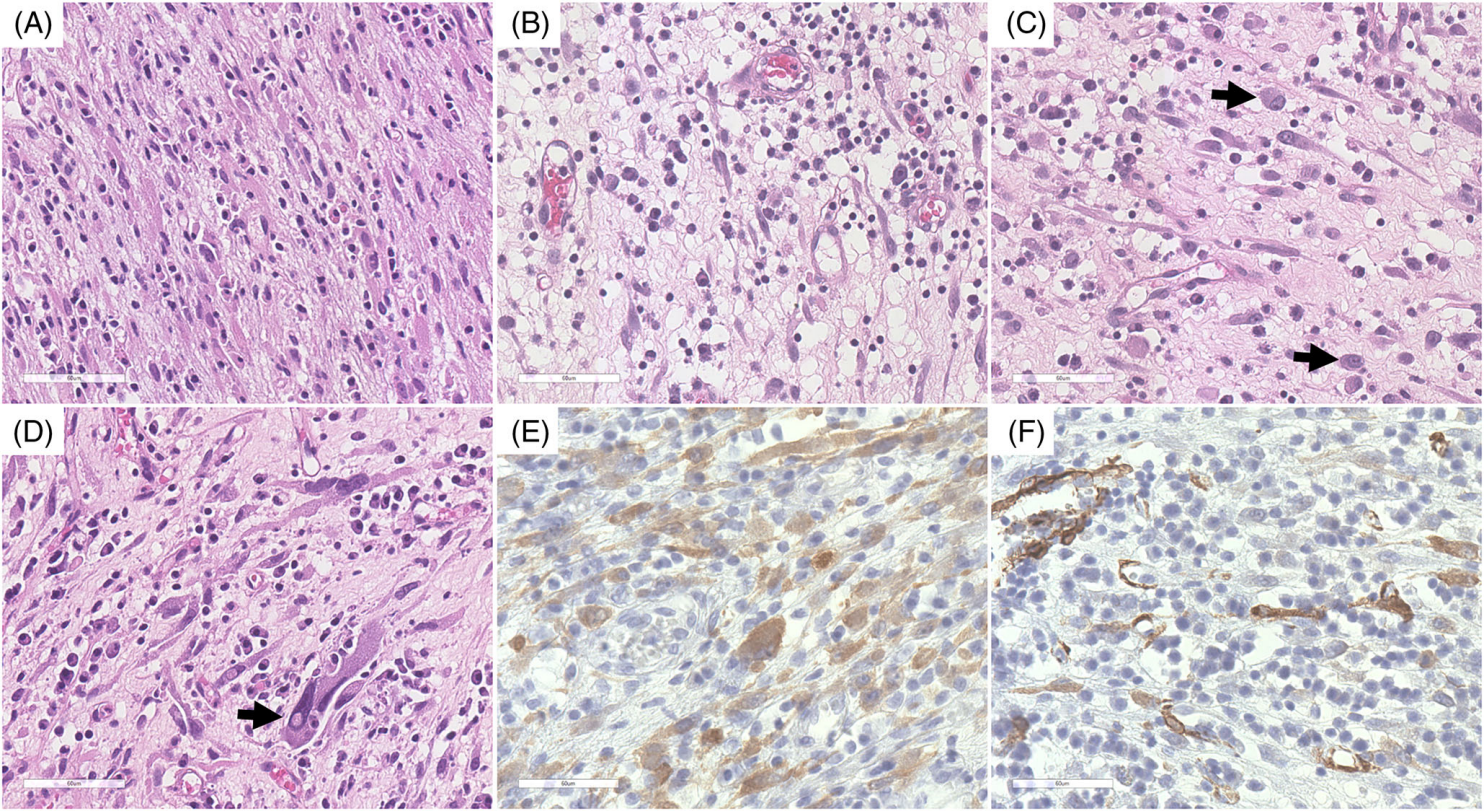
Histological and immunohistochemical features of the tumor. Spindle‐shaped cells with dense plasmacytic infiltration (A) and myxoid stroma (B) are observed. Ganglion‐like cells (C) and pleomorphism (D) are present. Spindle cells show diffuse ALK1 staining with heterogeneous cytoplasmic and focal nuclear expression (E) and focal actin positivity (F). ((A)–(D): H&E stain; (E), (F): Immunohistochemistry; original magnification ×300).

## 3 FINAL DIAGNOSIS

Inflammatory myofibroblastic tumor.

## 4 DISCUSSION

IMT, a spindle cell neoplasm of intermediate malignancy, long considered an inflammatory pseudotumor (IPT), has been recognized and described as a distinct entity in the 2020 WHO classification of soft tissue tumors [1]. Intracranial IMT is extremely rare, accounting for 21 out of 25 reported cases in the literature of central nervous system (CNS) IMT/IPT in patients aged 0–24 years [2]. Due to its rarity in the CNS, IMT is not included in the 2021 WHO Classification of CNS Tumors, which instead lists new entities with overlapping histological features [3].

Clinical and MRI features of IMTs are nonspecific, and diagnosis relies on histopathology and immunohistochemistry. Histologically, IMTs show spindle cell proliferation with dense lymphoplasmacytic infiltrate and three main patterns that may coexist: (1) nodular fasciitis‐like, (2) fibromatosis/fibrohistiocytic, and (3) desmoid‐like. Necrosis, fascicular or herringbone architecture, multinucleation, pleomorphism, ganglion‐like cells, and atypical mitoses indicate more aggressive variants [1].

Spindle cells variably express myoid markers such as smooth muscle actin and desmin, but are typically negative for glial markers, S100, CD117, and DOG1. ALK1 expression is detected in 40%–60% of cases, correlates with *ALK* fusion, and serves as both a diagnostic and predictive marker for ALK‐targeted tyrosine kinase inhibitor therapy [1, 2]. Its subcellular staining pattern varies with fusion partners [1]. *TPM4* is a frequent fusion partner in pediatric IMTs, typically producing diffuse cytoplasmic staining, as observed in our case [1]. In ALK‐negative IMTs, *ROS1* and *NTRK3* rearrangements are most common [1, 2].

Histological features of IMT are not pathognomonic and may mimic other entities in the CNS. Intracranial mesenchymal tumor with *FET:CREB* fusion and *CIC*‐rearranged sarcoma may exhibit overlapping features [3]. The presence of ganglion‐like cells, cellular pleomorphism, and a predominant plasmacytic infiltrate requires distinction from other entities such as gliomas, lymphoplasmacytic‐rich meningioma, IPT, histiocytosis, or autoimmune diseases. Careful evaluation of histological pattern and immunohistochemical profile, including ALK, as well as molecular assessments, are essential to differentiate IMT from these mimics.

Surgical resection is the primary treatment for CNS‐IMT. When not feasible or in recurrent/metastatic cases, corticosteroids, chemotherapy, radiotherapy, and ALK inhibitors may be used, with ALK inhibitors showing promising second‐line results [2]. ALK rearrangements are usually detected by immunohistochemistry or FISH. If negative, modern additional molecular testing (NGS panels, RNA‐seq) can detect false‐negative results or other alterations [1]. Comprehensive molecular profiling is therefore crucial for optimal decisions.

## 5 CONCLUSION

Intracranial IMTs are rare neoplasms with poorly understood tumorigenesis and nonspecific clinical and radiological features. Pathologists should be aware of the existence of IMT in the CNS and perform ALK immunostaining and, if necessary, molecular analysis to identify gene fusions. Proper identification of this entity and its molecular characteristics is crucial for diagnosis and therapeutic purposes.

## AUTHOR CONTRIBUTIONS

*Conceptualization*: FBV, TTT; *Data analysis and interpretation*: FBV, TTT, TMHL, BDM, HNT, THN, GDP; *Drafting*: TTT, TMHL, HNT; *Critical review/editing*: FBV; Supervision: FBV. All authors approved the final manuscript.

## PATIENT CONSENT

Informed consent was obtained from the patient's parents.

## CONFLICT OF INTEREST STATEMENT

The authors declare no conflict of interest.

## Data Availability

The data that support the findings of this study are available from the corresponding author upon reasonable request.
